# Reliability and validity of the “XingXun” system for measuring punch acceleration and velocity in elite boxers

**DOI:** 10.3389/fphys.2026.1726442

**Published:** 2026-02-20

**Authors:** Ji Qi, Rangxi Jin, Tongling Wang, Zengyi Li, Mitchell James Finlay, Chao Chen

**Affiliations:** 1 College of Physical Education, Harbin Normal University, Harbin, Heilongjiang, China; 2 School of Athletic Performance, Shanghai University of Sport, Shanghai, China; 3 Institute of Physical Education, Huzhou University, Huzhou, Zhejiang, China; 4 Sport Department, University Academy 92, Manchester, United Kingdom; 5 College of Physical Education, Dalian University, Dalian, Liaoning, China

**Keywords:** boxers, boxing, punch acceleration, punch velocity, reliability and validity

## Abstract

**Introduction:**

Punching velocity-related indicators are crucial in boxing, and accurately quantifying these metrics is significant for evaluating athletes’ performance. This study aims to assess the reliability and validity of a commercial inertial sensor-based boxing training monitoring system (“XingXun”) for measuring punch acceleration and velocity.

**Methods:**

Nine male boxers from the Shanghai University of Sport (age: 24.8 ± 3.1 years) with national-level competitive experience participated in the study. Participants wore “XingXun” sensors on both hands and performed maximum-effort tests involving six single punches (jabs, hooks, and uppercuts) and a 3-minute combination punch test. Concurrent validity was established by comparing the “XingXun” measurements against a 3D motion capture system (Qualisys) as the gold standard. Reliability was assessed using the Intraclass Correlation Coefficient (ICC) and Coefficient of Variation (CV). Validity was determined through Pearson correlation analysis, paired t-tests, Mean Deviation (MD), and Mean Absolute Error (MAE). Bland-Altman plots were used to visualize the agreement between the two systems.

**Results:**

The “XingXun” system demonstrated acceptable to excellent reliability for all punch types, with ICC values ranging from 0.883 to 0.950 and CV from 2.27% to 7.67% for acceleration and velocity. Validity analysis showed a strong correlation between the “XingXun” and Qualisys systems for both punch acceleration (r = 0.836–0.911) and velocity (r = 0.785–0.854). The measurement errors were small and not statistically significant (P > 0.05 or ES < 0.2), and Bland-Altman plots confirmed a high level of agreement between the two devices.

**Conclusion:**

The “XingXun” boxing training monitoring system is a reliable and valid tool for quantifying punch acceleration and velocity, offering a practical alternative to laboratory-based 3D motion capture for athlete performance monitoring.

## 1 Introduction

Boxing is an intermittent, high-intensity combat sport (Davis et al., 2018), that holds significant attention in both competitive and recreational sports. During a bout, athletes must inflict as much damage to an opponent via a knockout, technical knockout, or via an accumulation of strikes resulting in a decision victory. Punch force, velocity, and acceleration, are key indicators for effective punches (Loturco et al., 2016), though there are other variables of interest, and of course in some cases these variables are related. The quantification of said variables are of interest to researchers, practitioners, and combat athletes who may wish to analyse technique, profile athletes, or monitor the effectiveness of training programmes and specific interventions. However, the lack of effective monitoring methods for key parameters in boxing has long been a challenge for practitioners and coaches, limiting the precision of targeted training plans and impact on athletic and competitive performance. The quantification of punch impact force, whilst important in a sport where inflicting damage via a single or accumulated impacts is desirable, can be logistically challenging. Specifically, the cost of vertically mounted force plates and similar technology can be expensive, and typically limited only to laboratory settings (Finlay et al., 2021), thus the ecological validity may also be limited. Depending on the instrument and the research question, data processing and analysis methods may also be time consuming. With respect to punch velocity and acceleration measurement, there are two main methods. One method is vision-based, again typically used in laboratory settings, and involving high-frame-rate video analysis and 3D motion capture. These methods record visual data or 3D motion data to measure each punch’s velocity, position, acceleration, and momentum (Piorkowski et al., 2011; Mack et al., 2010; Atha et al., 1985). This approach can serve as a ‘gold standard’ for validating contemporary devices (Cheraghi et al., 2014; Meric Bingul, 2017), but much like the kinetic assessment mentioned above, its practical application in training and competitive environments is rather limited (Bauer et al., 2015). In recent years, researchers have made significant advancements in developing wearable inertial sensor technology for detecting human activities and measuring biomechanical performance parameters, including directly and indirectly measuring punch velocity and acceleration in combat sports (Kimm and Thiel, 2015; Stojsih et al., 2010; Lambert et al., 2018; Menzel and Potthast, 2021). This technology has also been applied to punch bags, whereby kinematic variables can be used to estimate punch force (Dinu and Louis, 2020), in the absence of vertically-mounted instruments mentioned above. This technology has been used for the specific purposes of combat sports, used to measure relevant indicators during practice (i.e., sparring, or punch bag/pad work). Commercially available systems often analyze data with proprietary software, presenting comprehensible results to users without complex analysis. This effectively circumvents the drawbacks of traditional measurement methods, which as mentioned, are often cumbersome, computationally complex, and not intuitive in data presentation. However, the unstructured, repetitive, and highly dynamic nature of boxing makes it difficult for inertial sensor systems to achieve accuracy in testing. Furthermore, the reliability of such devices are often not stated, and are often inferior to 3D-motion analysis (Omcirk et al., 2021). There in lies the question of practicality vs. accuracy and reliability, where a balance is desirable.

Therefore, a device that can provide objective data would greatly assist coaches in monitoring athletes’ performance (Omcirk et al., 2021); however, there are many considerations. Firstly, in terms of punch types: each punch involves the entire kinetic chain, including the legs, torso, and arms (Finlay et al., 2022). As a result, the movement trajectory, punch velocity, and punch acceleration vary depending on the punch type. Additionally, unlike jab punches, hooks and uppercuts do not occur purely in the sagittal plane (Bauer et al., 2015). These punches require higher dynamic computational capabilities from devices in 3D space. Secondly, although different devices may have unique user interfaces and various types of data, and despite the internal technology being quite similar (Espinosa et al., 2019; Peake et al., 2018), each device and its built-in software are set up for a specific purpose, and differences in specifications and algorithms can lead to variations in reliability and validity when measuring the same indicators across different brands or models of devices.

While the feasibility of using wearable sensor technology to measure punch velocity and acceleration in boxers has been validated, few studies have focused on the crucial aspects of the development process, namely the stability and validity of the measurements (Bassett Jr et al., 2012; Seshadri et al., 2019; Andreoni et al., 2017). Namely, the below issues are highlighted within the literature, though the authors note it is not the aim of the study to address all of these. (i) There is no consensus in the scientific literature regarding measurement protocols (i.e., the number of sensors used, sensor placement, or sensor characteristics); (ii) there is no consensus on the signal processing of data extracted from sensors in the literature, and algorithms are often not shared across the broader scientific community; (iii) many inertial sensor classification algorithms rely on external technologies, such as optical motion capture, or are specific to particular sensor suites.

Recently, a comprehensive boxing performance monitoring system (“XingXun” boxing training monitoring system) has been developed in collaboration between a wearable technology company, a research institution, and a national boxing team. This system has been developed using smaller inertial sensors combined with wearable wristbands, and features accelerometers, gyroscopes, and magnetometers. Likewise, these devices purport to offer punch data visualization, data export, and data analysis. At the time of writing, the system is currently being trialed within a national boxing team and regularly used in training scenarios. However, to date, the reliability and validity in measuring key indicators have not yet been verified. Therefore, this study aims to examine the accuracy and validity of a contemporary boxing performance monitoring system. To do this, punch performance as quantified by the devices will be compared to that of gold standard 3D motion capture.

## 2 Materials and methods

### 2.1 Experimental approach to the problem

To study the reliability and validity of the “XingXun” boxing training monitoring system, participants were instructed to perform six maximum effort punches of a single type (to minimize any sequence effects potentially caused by internal algorithms (Piorkowski et al., 2011)) and a 3-min free Combination Punch test. The punches included lead jab jab, lead hooks [LH], lead uppercuts [LUC], rear jab [RJ], rear hooks [RH], and rear uppercuts [RUC]. During the test, “XingXun” measured the end-point punch velocity and peak punch acceleration. During the measurement process, it was not clear how the algorithm of “Xingxun” calculated the strike acceleration and strike velocity metrics from the raw inertial sensor data. This study utilised Qualisys optical 3D motion capture system to conduct both single-punch and continuous-punch tests, simultaneously evaluating the reliability and validity of the “XingXun” boxing training monitoring system.

### 2.2 Subjects

Nine male boxers from the Shanghai University of Sport (age: 24.8 ± 3.1 years, weight: 74.2 ± 10.7 kg, height: 176.9 ± 5.8 cm) voluntarily participated in this study. All participants had at least 4 years of training experience and had competed in at least one national competition. None of the participants had any injuries that could have affected participation in the study. Prior to the study, participants were provided with information regarding the risks, benefits, and processes involved in the study. Finally, the participants provided written informed consent in accordance with the ethical guidelines of the Shanghai University of Sport Ethics Committee, and the Helsinki Declaration. The ethics approval number is 102772024RT050.

### 2.3 Procedures

The tests were divided into single-punch tests and 3-min continuous-punch tests, both conducted within one training session. During the single-punch test, participants were verbally instructed by the lead researcher regarding the type and number of punches. For the continuous-punch test, participants were informed beforehand that they must include the jab and rear jab, hook, and uppercut punches. The lead researchers provided start and stop signals to indicate when to begin and end the punching sequence. Prior to any punching assessment, the participants were led through a warm-up that included 3 min of slow jogging, 2 min of rope skipping, 2 min of boxing footwork drills, 5 min of dynamic stretching, and 3 min of focus mitt training.

**FIGURE 1:**
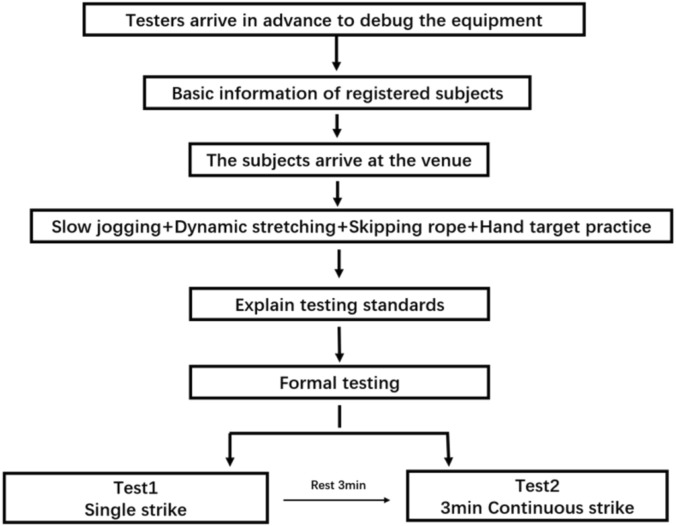
Testing flowchart.

Four sensors were attached to wristbands provided by the supplier (two sensors per hand, overlapped). The sensors were placed on the outer edge of the wrist, just above the distal radioulnar joint, and secured tightly to ensure they would not come loose during punches (Figure 1). Sensors were randomly numbered for each participant. During the development phase, Xingxun had already accounted for potential interference between multiple devices operating simultaneously, thus incorporating anti-interference features. The reason for overlapping identical sensors is to verify the reliability of the equipment under identical test conditions. Thanks to this design, the data collection and signal reception of the two overlapping sensors remain unaffected by each other.

Under the supervision of the researchers, participants engaged in a 3-min simulated test for both the single-punch and continuous-punch. During the simulated test, participants’ movements were monitored and corrected. This process served as a specific warm-up session and helps participants to get familiarized with the punching sequence, minimizing irregular punching trajectories that are unrecognizable by the sensor. This was preceded by a further familiarization trial whereby participants could become accustomed to punching with the devices, further minimising any potential learning effects in the study.

Single-Punch Test: Participants performed six maximal-effort punches of a single type in a controlled, static stance. Punches included Lead Jab (LJ), Lead Hook (LH), Lead Uppercut (LUC), Rear Jab (RJ), Rear Hook (RH), and Rear Uppercut (RUC). Each punch was performed with a brief pause (∼3 s) between repetitions to ensure maximal intent and avoid sequence effects.

3-Minute Continuous-Punch Test: Participants performed a simulated 3-min round of free combination punching. They were instructed to include all punch types (jabs, hooks, and uppercuts) in realistic sequences at self-selected intensity, approximating competitive pacing. Researchers provided start/stop signals.

### 2.4 Data acquisition and data analyses

#### 2.4.1 Data acquisition

**FIGURE 2:**
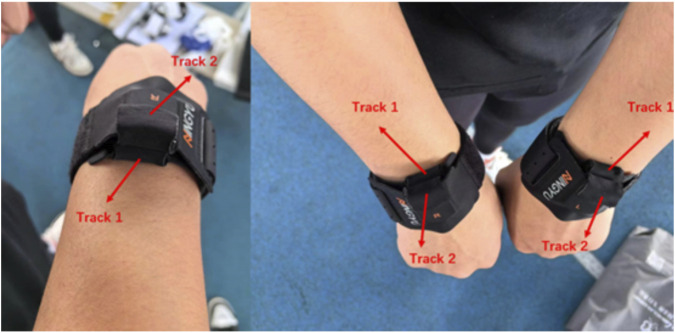
Example set-up of sensors.

The “XingXun” boxing punch monitoring device (BX260) used in this study may contain a microcontroller, accelerometer, gyroscope, and magnetometer, The inertial sensor is produced by TDK, with the model number ICM-20649 (Figure 2). However, the specifications of its measurements, data filtering procedures, data acquisition methods, and algorithms were not yet disclosed prior to the completion of this work. The data presented in this section and thereafter are not processed raw data by the researchers, but directly off the built-in application of XingXun. According to the operation manual of XingXun, the metrics adopted include end punch velocity and peak punch acceleration. The output data can be directly downloaded to an Excel.csv. file using built-in software. Punches that were not recorded due to technical failures are not compensated for in this study, with Table 1 providing the actual punch counts in detail. The raw 3D marker trajectory data from the Qualisys system were filtered using a low-pass Butterworth filter with a cut-off frequency of 15 Hz to reduce high-frequency noise while preserving the kinematic signal relevant to gross punch movements.

**TABLE 1:** Actual punch count observed in the study.

Punch type	Single punches measurement		Combination punches measurement	
	Sensor1 (n = 9)	Sensor2 (n = 9)	Sensor1 (n = 9)	Sensor2 (n = 9)
Lead jab	8.6 ± 1.0	8.5 ± 1.1	23.0 ± 3.8	23.1 ± 3.8
Lead hooks	9.1 ± 0.9	9.1 ± 0.8	13.1 ± 1.8	13.2 ± 2.0
Lead uppercuts	9.4 ± 0.7	9.3 ± 0.6	19.8 ± 3.1	19.9 ± 3.6
Rear jab	9.1 ± 0.5	9.0 ± 0.6	33.7 ± 5.5	33.6 ± 5.5
Rear hooks	8.9 ± 0.8	9.0 ± 0.7	14.6 ± 3.6	14.9 ± 4.0
Rear uppercuts	8.7 ± 0.7	8.7 ± 0.7	14.6 ± 3.7	14.6 ± 4.1

In addition to the boxing measurement system, the three-dimensional motion capture system used eight cameras (Oqus 7+ system, Qualisys Inc., Goteborg, Sweden) to measure the punch velocity and acceleration of athletes at a frequency of 500 fps. Following the guidelines provided by the manufacturer, the Qualisys infrared motion capture system was calibrated using a “T” calibration frame to establish the experimental movement range. Test data were recorded using Qualisys Track Manager (QTM) (Version 2.14, Qualisys Inc., Goteborg, Sweden), exported to a.csv. file using Qualisys Track Manager, and converted to Microsoft Excel for further analysis.

### 2.5 Data synchronization and signal processing

To ensure temporal alignment between the “XingXun” inertial measurement unit (IMU) system and the Qualisys optical motion capture system, a synchronized trigger protocol was implemented. At the beginning of each testing session, a researcher performed a distinct, rapid overhead clapping motion three times while wearing the IMU sensors. This motion generated a clear, simultaneous spike in the accelerometer data of all “XingXun” sensors and was also captured by the high-speed Qualisys cameras. The timestamps of these synchronized events were used to align the data streams from both systems offline, correcting for any inherent clock drift. All subsequent analyses were performed on these time-aligned datasets.

As noted, the proprietary algorithms of the “XingXun” system are not publicly available. However, based on the manufacturer’s documentation and the nature of the output metrics, the general processing pipeline can be inferred. The raw signals from the accelerometer and gyroscope are first calibrated and filtered (likely with a low-pass filter to remove high-frequency noise unrelated to gross motor patterns). The system then employs an event detection algorithm to identify the start and end of a punch. This is likely based on threshold crossings of the resultant acceleration or angular velocity, signifying the initiation of a rapid limb movement. Once a punch event is isolated, the system calculates the peak punch acceleration as the maximum value of the filtered resultant acceleration within that time window. The end punch velocity is calculated by integrating the acceleration trace over a defined terminal phase of the punch (or, as per the manual, averaging linear velocity over a short window after acceleration returns to baseline). The specific thresholds, filter cut-off frequencies, and integration windows constitute the intellectual property of the developers and were not accessible for this validation study.

### 2.6 Statistical analyses

After initial data processing, statistical analyses were performed using SPSS version 26.0 (IBM Corporation, New York, NY, USA) and GraphPad Prism 9 (GraphPad Software, US). The between-unit reliability of this study was primarily based on the Intraclass Correlation Coefficient (ICC) and the Coefficient of Variation (CV) of the test data from two sets of identical sensors under the same conditions. An ICC value greater than 0.9 indicates excellent reliability, 0.75–0.90 indicates moderate reliability, 0.5–0.74 indicates fair reliability, less than 0.5 indicates poor reliability, and below 0.1 is considered lack of consistency (Worsey et al., 2019). The CV was used to determine the absolute reliability of the data. When a CV is below 10%, the data are considered relatively stable. A CV between 10% and 20% indicates moderate variation in the data. A CV exceeding 20% suggests relatively high variation in the data (Dugan et al., 2004). Therefore, if the ICC is greater than 0.75 and the CV is less than 10%, the device may be deemed of acceptable reliability for the measured indicators.

Following confirmation of data normality, The normality of the test data was assessed using the Shapiro-Wilk test. To assess the relationship between “XingXun” measurements of velocity and acceleration and those of Qualisys, a Pearson correlation coefficient was calculated. Interpretation of the r values (Dugan et al., 2004) was as follows: very strong correlation (0.8–1.0), strong correlation (0.6–0.8), moderate correlation (0.4–0.6), weak correlation (0.2–0.4), and very weak or no correlation (0–0.2). Mean deviation (MD) and mean absolute error (MAE) were calculated to evaluate the measurement system. MD quantifies the directional bias (where MD > 0 indicates systematic overestimation and MD < 0 reflects underestimation relative to actual values), while MAE assesses the absolute magnitude of measurement errors to determine overall “accuracy evaluates” the size of the error. The formula for MAE is: MAE = 1/n Σ| measured value–standard value| (where Σ denotes summation and n represents the sample size). To assess potential significant differences between the two measurement methods, and the magnitude of any difference, a paired sample t-test was also conducted along with Cohen’s d effects size, calculated using the formula below:

$$d=\left(M1-M2\right)\;/\text{ SD pooled}$$

Where M1 and M2 are the means of XingXun and Qualisys, respectively, and SD pooled is the combined standard deviation between the two devices. Finally, a Bland-Altman plot was used to visually assess the mean bias, heteroscedasticity, and limits of agreement between the boxing measurement system, and the gold standard. When the coefficient of determination (r^2^) is greater than 0.1, it indicates the presence of proportional bias (Bartsch et al., 2012).

## 3 Results

### 3.1 Between-unit reliability

The between-unit reliability of different numbered sensors from “XingXun” under the same conditions is shown in Table 2. Different models of sensors demonstrated high consistency when measuring punching acceleration and velocity during Single Punches and consecutive punch tests under the same conditions (Punch acceleration: ICC = 0.871–0.950, CV = 3.2–6.2%; Punch velocity: ICC = 0.883–0.917, CV = 2.3–8.4%).

**TABLE 2:** Between-unit reliability data.

Punching method	Punch type	Punch acceleration		Punch velocity	
		ICC	CV	ICC	CV
Single punches	Lead jab	0.942	3.5%	0.905	2.6%
	Lead hooks	0.926	4.4%	0.883	2.6%
	Lead uppercut	0.936	3.2%	0.896	2.3%
	Rear jab	0.950	6.1%	0.916	3.5%
	Rear hooks	0.929	4.5%	0.902	2.3%
	Rear uppercut	0.939	3.3%	0.904	2.4%
Combination punches	Lead jab	0.887	4.7%	0.906	5.3%
	Lead hooks	0.871	5.2%	0.894	8.4%
	Front uppercut	0.879	5.9%	0.901	7.7%
	Rear jab	0.905	3.8%	0.917	6.6%
	Rear hooks	0.894	6.2%	0.898	5.7%
	Back uppercut	0.901	4.3%	0.909	3.9%

### 3.2 Validity

The correlation between the punching acceleration data measured by XingXun and the Qualisys data is shown in Table 3. XingXun demonstrated a strong correlation with Qualisys during the Single Punches and Combination Punches tests (Single Punches: r = 0.884–0.911, MD = −0.463–0.412, MAE = 2.50–3.14; Combination Punches: r = 0.880–894, MD = −0.813–0.841, MAE = 2.70–4.38). Notably, the correlation coefficients for different punch types in the Single Punches test were higher than those in the Combination Punches. In particular, the results for the Lead Jab (r = 0.901) and Rear Jab (r = 0.911) in the Single Punches test showed a strong correlation with that obtained via 3D Motion Capture. The results of the paired sample t-tests for punching acceleration are presented in Table 4. Except for the Lead Hooks (p = 0.005, <0.05; d = 0.11), Lead Uppercuts (p = 0.014, <0.05; d = 0.07), and Rear Hooks (p = 0.032, >0.05; d = 0.12) in the Combination Punches test, which exhibited significant differences compared to the values measured by Qualisys, all other punching methods and punch types showed no significant differences (p = 0.100–0.688, d = 0.01–0.07). Considering the large sample size, the effect sizes remained within an acceptable range although there were significant differences.

**TABLE 3:** Correlation test results of punching acceleration.

Punch type	Single punches			Combination punches		
	r	MD	MAE	r	MD	MAE
Lead jab	0.901**	0.229	2.96	0.880**	0.067	2.70
Lead hooks	0.884**	−0.463	3.14	0.836**	−0.813	3.54
Lead uppercuts	0.892**	0.188	2.50	0.859**	−0.647	4.38
Rear jab	0.911**	0.412	3.08	0.894**	0.095	2.88
Rear hooks	0.893**	−0.275	2.87	0.844**	−0.459	4.36
Rear uppercuts	0.898**	0.125	2.55	0.862**	0.841	3.55

MAE, Represent Mean Absolute Error; * represents significance at p < 0.05; ** represents significance at p < 0.01, MD, represent Mean deviation.

**TABLE 4:** Paired sample t-Test results of punching acceleration.

Punch type	Single punches			Combination punches		
	95% Confidence interval	P value	d	95% Confidence interval	P value	d
Lead jab	(-0.29, 0.75)	0.387	0.03	(-0.26, 0.39)	0.688	0.01
Lead hooks	(-1.01, 0.09)	0.100	0.07	(-1.37, -0.25)	0.005*	0.11
Lead uppercuts	(-0.26, 0.64)	0.408	0.03	(-1.16, -0.13)	0.014*	0.07
Rear jab	(-0.16, 0.99)	0.158	0.05	(-0.19, 0.38)	0.519	0.01
Rear hooks	(-0.79, 0.24)	0.293	0.04	(-1.51, 0.60)	0.390	0.05
Rear uppercuts	(-0.35, 0.60)	0.599	0.02	(0.08, 1.61)	0.032*	0.12

* represents significant difference at in acceleration between XingXun and Qualysis at p < 0.05.

The correlation between the punch velocity data measured by XingXun and Qualisys is shown in Table 5. In the Single Punches and Combination Punches tests, XingXun exhibited a strong correlation with Qualisys for all punch types, except for the Lead Hooks in the Combination Punches test (r = 0.785, MD = −0.11, MAE = 0.71). The punch velocity data collected for the other punch types showed a very strong correlation with Qualisys (r = 0.810–0.854, MD = −0.21–0.16, MAE = 0.51–1.06). The correlation coefficients for different punch types in the Single Punches test were higher than those in the Combination Punches test.

**TABLE 5:** Correlation test results of punching velocity.

Punch type	Single punches			Combination punches		
	r	MD	MAE	r	MD	MAE
Lead jab	0.839**	0.06	0.51	0.837**	0.01	0.54
Lead hooks	0.811**	−0.04	0.52	0.785**	−0.11	0.71
Lead uppercuts	0.827**	0.15	0.63	0.803**	0.05	0.92
Rear jab	0.854**	0.08	0.56	0.843**	0.06	0.66
Rear hooks	0.833**	−0.07	0.53	0.810**	−0.21	1.06
Rear uppercuts	0.837**	0.16	0.79	0.825**	0.07	0.67

* represents p < 0.05; ** represents p < 0.01.

The results of the paired sample t-tests for punch velocity are presented in Table 6. Except for the lead uppercut in the Single Punches test, which exhibited a significant difference compared to the values measured by Qualisys (p = 0.019; d = 0.12), all other punching methods and punch types showed no significant differences.

**TABLE 6:** Paired sample t-Test results of punching velocity.

Punch type	Single punches			Combination punches		
	95% Confidence interval	P value	d	95% Confidence interval	P value	d
Lead jab	(-0.04, 0.16)	0.236	0.05	(-0.05, 0.08)	0.716	0.01
Lead hooks	(-0.14, 0.07)	0.481	0.04	(-0.22, 0.01)	0.072	0.08
Lead uppercuts	(0.03, 0.28)	0.019	0.12	(-0.08, 0.17)	0.454	0.02
Rear jab	(-0.04, 0.20)	0.203	0.06	(-0.01, 0.12)	0.075	0.04
Rear hooks	(-0.18, 0.05)	0.253	0.06	(-0.45, 0.04)	0.096	0.11
Rear uppercuts	(-0.01, 0.32)	0.060	0.09	(0.09, 0.23)	0.396	0.05

Mean Difference represents the average value of the difference between XingXun and Qualisys.

### 3.3 Bland-AltmanAnalysis

**FIGURE 3:**
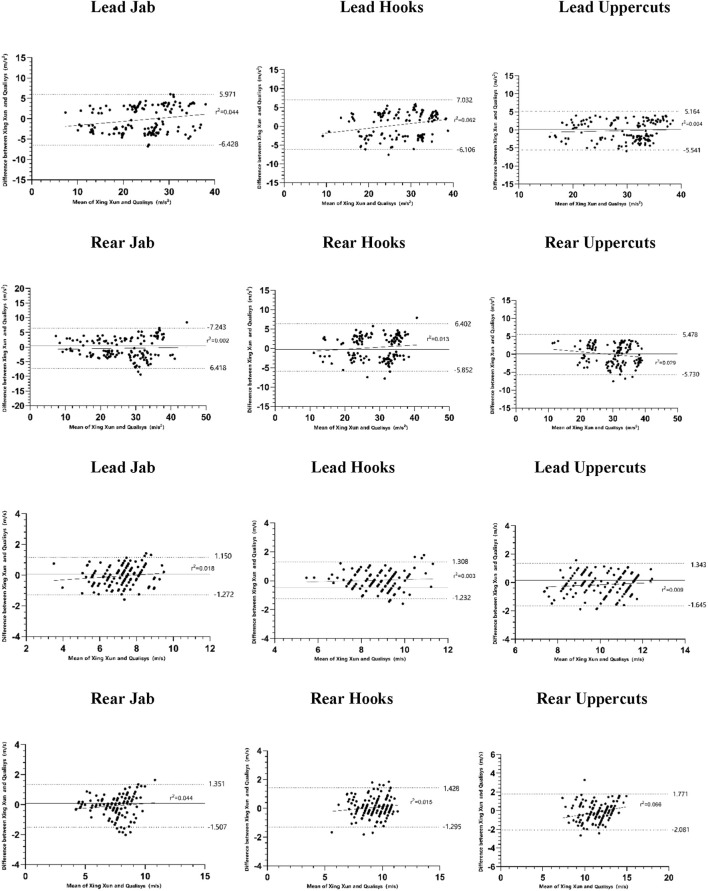
Bland-altman plot of single punches acceleration and velocity.

**FIGURE 4:**
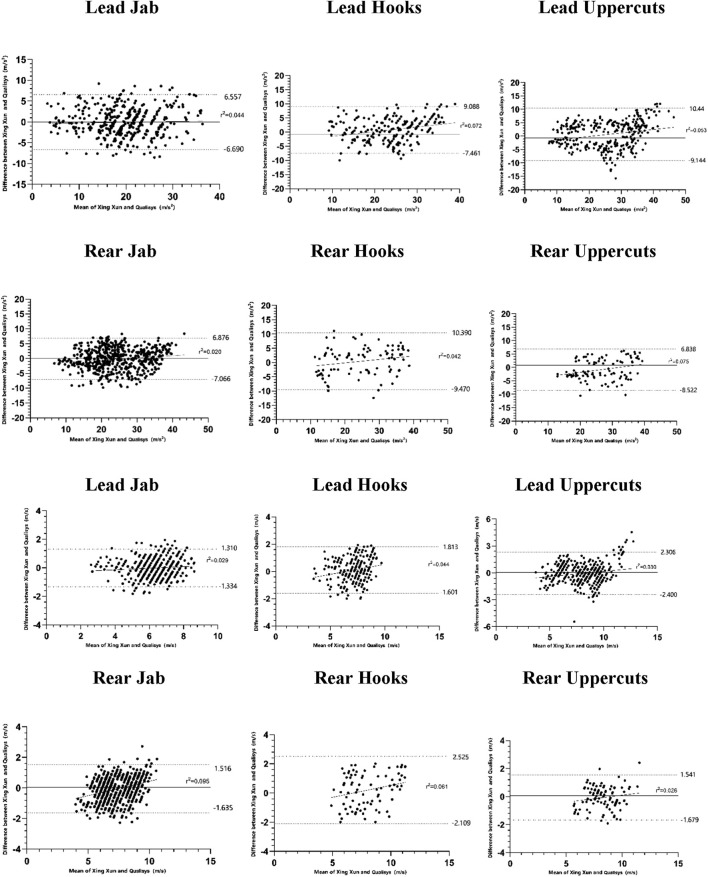
Bland-Altman plot of combination punches acceleration and velocity. Note: The solid line represents the difference and mean value between the peak acceleration (m/s^2^) and end punching velocity measured by “XingXun” and that from Qualisys. The dashed line indicates the limits of the 95% agreement interval, while the solid line shows the mean difference and magnitude of bias between the “XingXun” and Qualisys measurements.

Bland-Altman plots were generated to assess the agreement between the “XingXun” system and the Qualisys gold standard for both acceleration and velocity across all punch types (Figures 3, 4). The mean bias (solid line) for punch acceleration ranged from −0.46 to +0.41 m/s^2^ in single punches and from −0.81 to +0.84 m/s^2^ in combination punches. For punch velocity, mean bias ranged from −0.07 to +0.16 m/s. The 95% limits of agreement (dashed lines) were narrow for most punch types, indicating acceptable agreement. No consistent heteroscedasticity was observed across plots (r^2^ < 0.1 for most comparisons), suggesting that measurement error was not proportional to the magnitude of the measured values. Overall, the Bland-Altman analysis supports the conclusion that the “XingXun” system exhibits good agreement with the criterion measure.

## 4 Discussion

### 4.1 Potent

The purpose of this study was to explore whether the “XingXun Boxing Training Monitoring System” can provide valid and reliable data on punch acceleration and velocity in professionally trained boxers. In this study, we purposefully collected data from a set of sensors, numbered differently, taking simultaneous measurements during identical punches. We also compared the results of “XingXun” with those from 3D motion capture across all punch types.

A pivotal methodological consideration in this study is the distinction between algorithmic transparency and empirical validity. The proprietary nature of the “XingXun” system means its internal algorithms—the specific filtering techniques, event detection thresholds, and mathematical operations that transform raw inertial data into the metrics of “peak acceleration” and “end velocity”—are not disclosed (a black-box). However, this does not preclude the validation of its outputs. The system presents itself as a tool that provides specific, defined metrics. Our study therefore adopted a criterion-based validation approach. We treated the system as a unified measurement instrument: given a standardized physical input (a boxer performing a punch), does it produce an output that agrees with a gold-standard measure of the same physical phenomenon? By rigorously comparing its final output values against those from a 3D motion capture system across a wide range of punches and intensities, we assess its empirical validity for practical use. This approach validates the system’s utility as a ready-to-use coaching tool, even in the absence of full algorithmic transparency, which is a common scenario with commercial sports technology.

The main findings of this study are that the XingXun devices can be considered valid and reliable in monitoring punch velocity and acceleration in highly trained boxers. As such, the practitioner, coach, and athlete may confidently use the devices to monitor kinematic data relating to punch performance. The validity and reliability did vary across punches, and this is discussed in detail, later in this section.

### 4.2 Between-unit reliability

For isolated punch velocity and acceleration, good-to-excellent (ICC = 0.883–0.916), and excellent (ICC = 0.926–0.950) between-unit reliability was observed across different punch types. ICC’s were consistently lower for punch acceleration (ICC = 0.871–0.905) in combination tests, compared to isolated punches, though the same was not observed for velocity, with ICC values of (ICC = 0.894–0.917), very similar to that seen in isolated punches. The CV% was consistently higher in combination punches compared to isolated punches, suggesting that the absolute reliability of the data was weaker during combination punches. This can be expected due to the more dynamic nature of combination punches, inclusive of preparatory actions prior to the punch itself. Other factors that may impact this, could be system error caused by the sensors of different serial numbers, and sensor parameter issues. To measure such high-intensity actions, sufficiently high sampling frequencies, sampling ranges, hardware setting, and specific algorithms of the sensors are critical requirements. The participants in this study were amateur boxers. As shown in Table 2, the peak punching accelerations and velocities recorded are consistent with the high-performance ranges reported for athletes of this caliber in the literature (Dinu and Louis, 2020; Liu et al., 2022). This validates that the ‘XingXun’ system was tested under demanding kinematic conditions. Consequently, the high intensities achieved may have posed a challenge for the device’s specified 200 Hz sampling frequency during rapid sequences, potentially explaining the slightly wider limits of agreement observed in combination punches.”

### 4.3 Validity

In terms of validity, internal factors such as sampling frequency, athletes’ punching ability, and methods of raw data processing exert critical impact on the validity of punch acceleration measurements. The device being explored in the current study, “XingXun”, conducts punch acceleration measurements based on end punch velocity values. An analysis of punch acceleration measurement by “XingXun” reveals that it showed very high reliability and stability (r = 0.884–0.911) in Single Punches tests. While “XingXun” also demonstrated high accuracy in acceleration measurements during Combination Punches tests (r = 0.836–0.894), reliability across different punching types witnessed various degrees of decline. Such decline may be due to its own sampling frequency and the athletes’ performance levels. Firstly, as mentioned, the sampling frequency of “XingXun” is 200Hz, which meets the minimum standards identified in prior research for using inertial sensors in analyzing combat sports performance (Worsey et al., 2019). Secondly, the subjects in this study were professional athletes with significant competitive experience. As a result, their acceleration was relatively higher, and the frequency of punches much greater within a given time to the point that a 200 Hz sampling frequency may not be adequate. This led to reduced reliability and validity during Combination Punches tests. In addition, as mentioned previously, uppercuts and hook punches involve movements in three dimensions compared to jab punches, making measurement even more challenging or the sensors to detect and quantify.

Since “XingXun” collects peak acceleration values, it can better reflect actual athletic performance especially at lower sampling frequencies (Dugan et al., 2004). Although the validity of punching acceleration measurement during Combination Punches tests slightly decreased, it remained within a good range. Due to the rapid rotation and high-impact nature of striking, data processing methods can influence the reliability and validity of systems. Matthew et al., reviewed the use of inertial sensors in combat sports, and reported inconsistency in the reporting of exact calculations used to define acceleration values. Since “XingXun” has not published its raw data processing algorithms, this study only employs common methods for calculating acceleration metrics using inertial sensors. It is speculated that data processing methods may have a major impact on the reliability and validity of “XingXun” system, without further discussion on computational logic. Therefore, sampling frequency, performance level of the athletes, and data processing methods of “XingXun” could all contribute to the decreased accuracy in acceleration measurements.

“XingXun” defines the measured punch velocity as the end velocity, specifically the average linear velocity of the first five frames when the punching end acceleration is 0 m/s^2^. Qualisys calculates this at the same time point to achieve data alignment. This study finds that “XingXun” demonstrates good validity in measuring punching velocity. For punch velocity, a statistically significant difference was found between systems for the Lead Hook during single punches (p = 0.019). However, the effect size for this difference was small (d = 0.12), and for all other punch types across both single and combination tests, no significant differences were observed (p > 0.05), with effect sizes ranging from negligible to small. Therefore, considering the large sample size and the predominant pattern of small, non-significant differences, the “XingXun” system can be considered to demonstrate good concurrent validity for measuring punch velocity. This observation, along with Table 5 (Single Punches: MAE = 0.51–0.79; Combination Punches: MAE = 0.51–1.06), suggests that measurement errors increase at faster punching velocitys. One possible reason is that “XingXun” does not allow any additional input that may influence punching velocities, such as fist size and arm length, aside from the athlete’s height, weight, and stance type. This means that the software must have used certain predefined values, which may affect measurements since punching velocity is reported as linear velocity, and athletes’ arm span directly impacts the measurement of punching velocity (McGinnis, 2013). This is consistent with the views presented by Dan et al. (Omcirk et al., 2021). Additionally, any alteration in the sensor placement, even if the user follows the manufacturer’s guidelines, can reduce the validity of the research measurements. On the other hand, the accuracy of punching velocity measurement also depends on the accuracy of the collected punching acceleration, and errors in acceleration measurements can further impact the accuracy of punching velocity measurements.

A notable finding was the statistically significant difference in acceleration for some punches (e.g., hooks and uppercuts) during combination tests, contrasted with the consistent agreement for velocity across all punches. This discrepancy likely stems from fundamental differences between the metrics adopted include peak punch acceleration and end punch velocity. The ‘XingXun’ system processes raw inertial sensor data through proprietary, undisclosed algorithms to first identify a punch event and then output these specific metrics. According to the manufacturer’s operation manual, the system’s calculation of end punch velocity is defined as the average linear velocity over the first five frames once the hand acceleration returns to zero at the punch endpoint. The peak punch acceleration is the maximum resultant acceleration detected during the identified punching motion. It is important to note that the precise algorithmic steps, filtering parameters, and the exact computational relationship between the raw signals and these two output metrics are not disclosed by the manufacturer.

This observed discrepancy, where acceleration shows differences but velocity does not—even though velocity is derived from acceleration—can be explained by the mathematical properties of signal integration. The process of integrating acceleration to obtain velocity inherently smooths out high-frequency noise and minor, non-systematic timing errors that can significantly affect the instantaneous peak value (acceleration). In other words, random variations in acceleration tend to cancel each other out over the short integration window used to calculate endpoint velocity. Therefore, the velocity metric emerges as a more robust and stable output, consistent with our finding of high agreement, despite greater sensitivity in pinpointing the exact peak acceleration.

Overall, the main findings are as follows: (A) “XingXun” shows good results in measuring punching acceleration and velocity for different punching types in both Single Punches and Combination Punches tests; (B) the reliability and validity of “XingXun” in measuring jab punches are more stable and accurate than those for uppercuts punches and hook punches; (C) increasing punching frequency affects the stability and accuracy of “XingXun” in measuring punching acceleration and velocity, primarily due to the lower sampling frequency.

### 4.4 Practical recommendations

The logical starting point for developing the “XingXun” Boxing Training Monitoring System is to accurately and reliably monitor key indicators during the punching process. This study examined retest reliability and criterion validity of “XingXun.” The results indicate that “XingXun” is a reliable device for measuring punching acceleration and velocity. Coaches and athletes who are in need of a device that can measure both punching acceleration and velocity while recording punch can confidently consider “XingXun.” Furthermore, users should be aware that the stability and validity of Single Punches tests are higher than that of Combination Punches, and the faster the punching velocity, the greater the risk of measurement errors. The “XingXun” Boxing Training Monitoring System also provides indicators such as punching force and reaction time. Due to experimental limitations, this study focused only on the reliability and validity of punching acceleration and velocity. Future study may focus on the validity and reliability of other metrics measured by “XingXun,” and comparative studies may be conducted as a function of training environment.

### 4.5 Limitations

This study is not without limitations. A key limitation stems from the proprietary and specialized nature of the “XingXun” system. First, as a custom-built, research-oriented device developed in collaboration with a national team, it is not a commercially available consumer product, which may limit immediate access for other researchers and practitioners. Second, the exact computational logic for deriving punch acceleration and velocity from the raw IMU data is not disclosed. While this lack of algorithmic transparency prevents a deep biomechanical dissection of the signal processing chain, our study focused on a criterion-based validation approach. By comparing the system’s final output against a gold-standard, we assessed its *empirical validity* for providing ready-to-use performance metrics.

## Data Availability

The raw data supporting the conclusions of this article will be made available by the authors, without undue reservation.
